# Locus Coeruleus atrophy doesn’t relate to fatigue in Parkinson’s disease

**DOI:** 10.1038/s41598-018-30128-y

**Published:** 2018-08-17

**Authors:** Oleg Solopchuk, Moustapha Sebti, Céline Bouvy, Charles-Etienne Benoit, Thibault Warlop, Anne Jeanjean, Alexandre Zénon

**Affiliations:** 1 Institute of Neuroscience, Université catholique de Louvain, Brussels, Belgium; 2 Cliniques Universitaires Saint Luc, Université catholique de Louvain, Brussels, Belgium; 3 INCIA, 33076 Bordeaux, France

## Abstract

Fatigue is a frequent complaint among healthy population and one of the earliest and most debilitating symptoms in Parkinson’s disease (PD). Earlier studies have examined the role of dopamine and serotonin in pathogenesis of fatigue, but the plausible role of noradrenalin (NA) remains underexplored. We investigated the relationship between fatigue in Parkinsonian patients and the extent of degeneration of Locus Coeruleus (LC), the main source of NA in the brain. We quantified LC and Substantia Nigra (SN) atrophy using neuromelanin-sensitive imaging, analyzed with a novel, fully automated algorithm. We also assessed patients’ fatigue, depression, sleep disturbance and vigilance. We found that LC degeneration correlated with the levels of depression and vigilance but not with fatigue, while fatigue correlated weakly with atrophy of SN. These results indicate that LC degeneration in Parkinson’s disease is unlikely to cause fatigue, but may be involved in mood and vigilance alterations.

## Introduction

**Figure 1:**
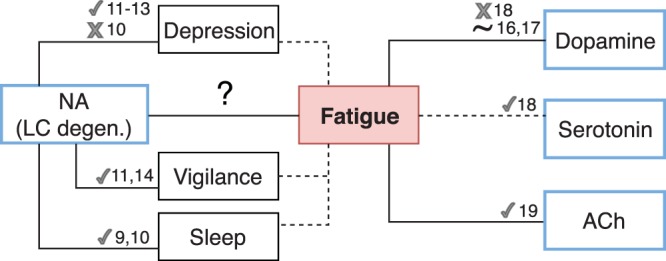
Schematic illustration of the possible neural correlates of fatigue. The numbers correspond to the relevant references. Neurotransmitters are highlighted in blue; ‘v’, ‘x’ and ‘~’ signs refer to positive, negative and inconclusive evidence in favor of a certain link.

Fatigue is widespread among healthy subjects and can become incapacitating in a number of neurological conditions including stroke, traumatic brain injury, multiple sclerosis and Parkinson’s disease (PD)^1^. In PD, fatigue appears years before motor disturbances and diagnosis, and is one of the three most disabling symptoms^1^. Nevertheless, it remains largely under-recognized by clinicians^2^, mostly due to our limited understanding of its neurobiological mechanisms (Fig. 1).

Strikingly, the possible implication of noradrenergic dysfunction in fatigue has not been thoroughly investigated. Noradrenaline (NA) is a neuromodulator that plays an important role in the modulation of cognition^3^, most notably known for its effect on behavioral and cortical arousal, as well as on sleep-wakefulness cycle^4^. Most NA producing cells in the brain cluster in LC^5^ and send numerous widespread projections to virtually all brain regions. Importantly, it has been known for long that LC degenerates among PD patients by up to 70% of its size^6^ and that NA concentrations in PD decrease to a comparable extent^7^. Similarly to pathogenesis of fatigue, such degeneration occurs very early in the evolution of PD, and was even shown to precede dopaminergic denervation^8^. Previously, LC atrophy in Parkinson patients has been frequently associated with non-motors symptoms such such as percentage of Rapid Eye Movement sleep without atonia^9,10^, depression^11–13^ and vigilance^11,14^.

Here, we hypothesized that fatigue encountered by PD patients could be related to LC degeneration. We tested this hypothesis on PD patients in various stages of disease progression, quantifying the extent of LC degeneration with neuromelanin-sensitive magnetic resonance imaging (MRI) and symptoms such as fatigue, depression, vigilance and sleep disorder by means of psychometric and behavioral assessment.

## Results

We investigated the relationship between LC degeneration and fatigue in PD, while also considering patients’ clinical characteristics using a battery of questionnaires (Supplementary Table 1). We found that fatigue correlated significantly with level of depression (PFS-BDI, r_K_ = 0.401, Bayes Factor (BF): 77.35, p < 0.001) and sleep disturbance (PFS-PDSS2, r_K_ = 0.318, BF: 8.674, p = 0.007) but not with general PD severity or levodopa-equivalent dose (LED; all p > 0.05, Table 1). We also found weak positive associations between level of depression and both sleep disturbance (BDI - PDSS2 r_K_ = 0.248, p = 0.040, BF: 1.923) and general disease severity (BDI – MDS-UPDRS, r_K_ = 0.267 p = 0.039, BF: 1.966).

**Table 1:** Correlation table, showing the relationships between questionnaires, disease severity and levodopa – equivalent doses (upper panel) and between fatigue (PFS), depression (BDI), sleep disturbance (PDSS2), disease severity (MDS-UPDRS total) and LC/SN degeneration (lower panel).

*BF_10_ > 5, **BF_10_ > 10, ***BF_10_ > 50		Depression (BDI)	Sleep (PDSS2)	Disease (MDS-UPDRS)	Medication(LED)
Fatigue (PFS)	Kendall’s tau	0.401***	0.318*	0.223	0.125
	BF_10_	77.35	8.674	1.072	0.37
	p-value	< 0.001	0.007	0.077	0.331
Depression (BDI)	Kendall’s tau		0.248	0.267	0.089
	BF_10_	—	1.923	1.966	0.293
	p-value		0.04	0.039	0.494
Sleep (PDSS2)	Kendall’s tau			0.24	0.136
	BF_10_		—	1.371	0.406
	p-value			0.059	0.29
Disease (MDS-UPDRS)	Kendall’s tau				0.323
	BF_10_			—	4.405
	p-value				0.015
*BF_**10**_ > 5, **BF_**10**_ > 10, ***BF_**10**_** > **50		**Fatigue (PFS)**	**Depression (BDI)**	**Sleep (PDSS2)**	**Disease (MDS-UPDRS)**
Locus Coeruleus	Kendall’s tau	0.061	−0.306*	0.171	−0.085
	BF_10_	0.248	5.104	0.588	0.291
	p-value	0.609	0.012	0.161	0.509
Substantia Nigra	Kendall’s tau	−0.281	−0.139	−0.018	−0.039
	BF_10_	3.35	0.421	0.224	0.264
	p-value	0.018	0.258	0.882	0.762

The BF_10_ represents the relative probability of the data under alternative hypothesis H1 with respect to null hypothesis H0 (i.e. BF_10_ of 20 indicates that the data is 20 times more likely to be observed under the alternative hypothesis).

Additionally, subjects performed an auditory oddball detection task to assess their levels of vigilance, or ability to maintain attention over time. Vigilance is commonly measured as performance decrement over time during monotonous tasks^15^. Here we assessed it by looking at the increase in RT with time-on-task. As expected, we found that reaction time (RT) increased overtime (t-test on RT slopes: p = 0.01). Neither mean RT nor change of the RT overtime correlated with any of the questionnaires completed by the patients (all p > 0.05, all BF < 0.79).

**Figure 2:**
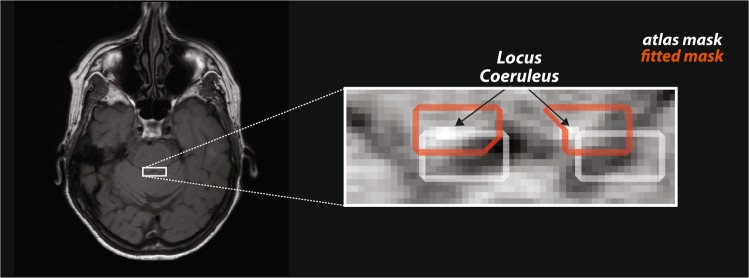
Schematic illustration of Locus Coeruleus isolation. We normalized the whole brain anatomical image (not shown) to the atlas template, and then projected the location of LC defined in atlas space to the individual neuromelanin-sensitive image (left), followed by an additional precision-fitting step. Afterwards, the LC index was computed from the voxels with high signal intensity (extracted with the unsupervised k-means algorithm, see Methods). The masks for all subjects can be found in the Supplementary Fig. 1.

**Figure 3:**
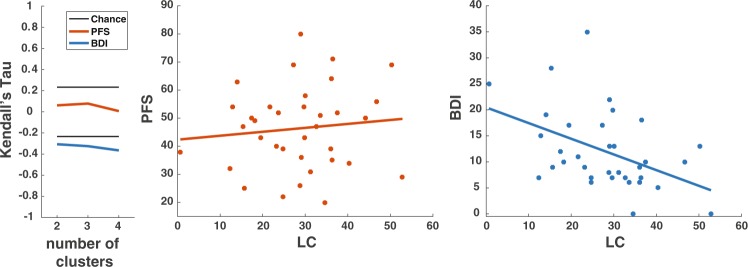
Relationship between LC degeneration, fatigue and depression. Leftmost panel: correlation coefficients for different K of the k-means algorithm for LC isolation: sensitivity of the algorithm did not depend on the number of clusters. Middle panel: scatterplot of LC index (K = 2) and fatigue measured with PFS. Rightmost panel: scatterplot of LC index (K = 2) and depression measured with BDI.

Regarding the analysis of LC size (Fig. 2), we found that its intensity (i.e. reciprocal of estimated degree of degeneration) correlated with depression (LC-BDI, r_K_ = −0.306, p = 0.012, BF: 5.104, Fig. 3) but not with fatigue (LC-PFS, r_K_ = 0.061, p = 0.609, BF: 0.248, decisive evidence for H_0_) or sleep disturbance (r_K_ = 0.171, p = 0.162, BF: 0.588). LC degeneration correlated also with both measures of vigilance: mean reaction time and change overtime (LC-RT mean, r_K_ = −0.351, p = 0.003, BF: 15.46; LC-RT change, r_K_ = −0.321, p = 0.006, BF: 7.670), indicating that patients with the strongest LC degeneration had the slowest average reaction time and the highest RT increase over time.

We performed two additional exploratory analyses. First, we quantified the degeneration of the Substantia Nigra (SN, see Methods, Supplementary Fig. 2), and found that, in contrast to LC, SN degeneration correlated weakly with fatigue scores (SN-PFS, r_K_ = −0.281, p = 0.018, BF: 3.350, weak evidence for H1). Second, we attempted to determine whether pupil response to oddball target detection (Supplementary Fig. 3) could be used as a functional measure of LC degeneration. We found, surprisingly, that pupil responses correlated strongly with SN, but not LC degeneration: linear mixed model with Block, SN intensity and LC intensity as factors highlighted a significant influence of SN (p = 0.003, estimate = 0.014, 95%CI: [0.005, 0.023]), Block (p = 0.043, estimate = −0.070, 95%CI: [−0.138, −0.002]), but not LC (p = 0.090, estimate = −0.016, 95%CI: [−0.035, 0.003]).

## Discussion

**Figure 4:**
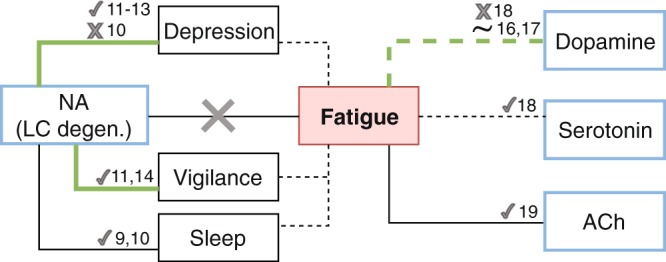
Schematic illustration of how our positive (in green) and negative (crossed) findings integrate with the literature. The link between Fatigue and Dopamine is dashed as it was weak, and found in an exploratory analysis.

In this study, we explored the plausible and under-investigated link between degeneration of LC, the main source of cerebral NA, and fatigue in PD. We found that LC degeneration was associated with depression and vigilance loss but, contrary to our predictions, not with fatigue (Fig. 4). In contrast, in a post-hoc analysis, we found weak evidence for a link between fatigue score and SN degeneration.

The relationship between neuromodulatory systems, fatigue, depression and sleep disturbance is considerably intricate. There is inconsistent evidence for the role of dopaminergic system in PD fatigue pathogenesis^1,16–18^, and some studies suggest a possible involvement of cholinergic^19^ and serotoninergic^18^ pathways. Depression is a frequent symptom among PD patients^20^, and is linked with severity of LC degeneration, as found in the present, and earlier studies^12,13^. However, fatigue often remains unrelieved following either dopaminergic replacement therapy^21^ or anti-depressant treatment^22^, suggesting different pathophysiological mechanisms for motor, mood and fatigue symptoms. Like depression, disturbance of sleep is highly debilitating for PD patients^23^, and in our study we also found a link between severity of fatigue and sleep disorders. However, sleep does not always have restorative effect on fatigue and patients complain of fatigue even in the absence of sleep disorder^1,24^.

It is also worth to mention several limitations of our study. First, fatigue assessment was based on psychometric methods, and was therefore submitted to the usual limitations of this approach. Notably, fatigue assessment through self-questionnaires can be confounded with similar concepts such as sleepiness or depression^25^. This cross-influence was partly accounted for in our study, in which we included separate questionnaires for these three concepts. Second, our estimates of LC and SN degeneration were based only on voxel intensities, neglecting the spatial extent of the structures. It remains currently unclear which approach provides the best estimate of degeneration. Third, the relationship between neuromelanin content and NA denervation requires careful considerations. It has been shown that the number of cells that contain neuromelanin (a byproduct of catecholamine synthesis) is roughly the same as the number of LC cells that contain tyrosine hydroxylase (a critical enzyme used for catecholamine production), at least in older adults^26,27^. These findings suggest, albeit quite indirectly, that presence of neuromelanin is specific to cells that produce NA and that reduction in neuromelanin signal reflects degeneration of NA-producing cells. Finally, we only tested the patients while they were on the dopamine replacement therapy. Despite the fact that non-motor symptoms of Parkinson’s disease such as fatigue and depression are notoriously resistant to medication^1,28^, it would be interesting to also test the patients off-treatment in a future study.

In our additional exploratory analyses, surprisingly, we found a relationship between SN degeneration and pupil responses, while the correlation with LC was absent. This finding appears to contradict earlier claims that have emphasized the link between LC activity and pupil responses^29^. However, the causality of the LC-pupil relationship has been put into question recently^30^ and the present results are the first to address the effect of LC degeneration on pupil size. Additionally, compensatory mechanisms could very well intervene to countervail the loss of noradrenergic neurons. For instance, the remaining population (or other NA producing nuclei^31^) could increase its average discharge rate to maintain overall cortical concentration of NA unchanged. The correlation of pupil responses with SN degeneration is also surprising given that activity of neurons in SN pars compacta was shown to be unrelated to pupil responses in primates^32^. However, the complex interrelation between dopamine and NA systems^33^ suggests that SN degeneration may impact on pupillary responses through its effect on general arousal mechanisms. These puzzling findings will require further investigation. Similarly, we found that SN degeneration correlated with fatigue, a link that may be worth exploring in a future study, despite the lack of cohesion in the literature^16–18^. At the same time, SN signal intensity failed to correlate with disease severity. This might seem surprising, however, previous findings are highly controversial, with some reporting significant correlations^34^ while others failing to find significant effect^35,36^. The latest results highlight the importance of spatial specificity of SN measurement, as disease severity was correlated only with subparts of SN^37^, or only with SN contralateral to the clinically impaired body side^38^.

All in all, we found that the decrease in neuromelanin signal in LC is not related to the severity of fatigue but is associated with increased level of depression and decreased vigilance. Elucidating the underlying mechanisms of fatigue will have a major impact on development of pharmacotherapy to improve patients’ quality of life and we believe that our study will advise further research on the neural correlates of fatigue.

## Methods

The experiment comprised one unique session, divided in two parts, whose order was counter-balanced across subjects. One part consisted of the MRI acquisition and the other part corresponded to the auditory oddball detection task. For each patient, we fine-aligned the LC mask derived from atlas (see Methods). Afterwards, in order to isolate high intensity region of LC, we applied a k-means clustering algorithm on the intensity of voxels within the aligned atlas region (Fig. 2, Supplementary Fig. 1). The cluster with the highest intensities was assigned to LC and the values of the cluster were normalized with respect to mean brainstem intensity^39^ to compute LC index.

### Subjects

Thirty-eight patients took part in this study (21 M/17 F, 38–75 years old), after providing their informed consent. All experimental procedures were approved by the local ethical committee (Comité d’Ethique Hospitalo-Facultaire Saint-Luc UCL) and conducted according to the declaration of Helsinki. The participants were diagnosed with idiopathic PD, from low to moderate severity (MDS-UPDRS total score: 53.1 ± 23.5, H&Y: 1.93 ± 0.69; mean ± sd), and were undergoing dopamine replacement therapy. Disease severity was investigated using the Unified Parkinson’s Disease Rating Scale, MDS-UPDRS^40^, fatigue with the Parkinson Fatigue Scale, PFS^41^, mood with the Beck Depression Inventory, second edition, BDI-II^42^ and sleep quality by means of Parkinson’s Disease Sleep Scale, PDSS-2^43^ questionnaires. All evaluations were performed under dopamine replacement treatment. For each patient, we also calculated levodopa equivalent doses (LED) of medication^44^. Due to the lack of data on safinamide LED, the safinamide doses were not integrated in the calculation of the total LED for 2 patients. Additionally, MDS-UPDRS, age, duration of the disease and LED were not assessed for 5 patients; PFS, BDI, PDSS for 3 patients, and the scanning was not performed for 2 patients due to contraindications revealed on the day of the experiment. The datasets are available from the corresponding author on reasonable request. All statistical analyses were performed using JASP 0.8.2 (JASP Team, 2017) and MATLAB (The MathWorks, Natick, MA, USA). Correlations between variables were performed with Kendall correlation in order to mitigate the influence of outliers and non-normality of LC index distribution (Fig. 3B,C), and are reported along with the Bayes factors (BF).

### Oddball detection task

During this task, series of tones were presented through the loudspeakers. Participants were seated in front of a 19″ CRT screen cadenced at 100 Hz, with their head resting on a chinrest. They were instructed to respond as quickly as possible to target tones (1000 Hz) by a mouse click, while ignoring the standard tones (500 Hz). Targets were randomly interspersed throughout the task such that 4 to 10 standard tones (drawn from a uniform distribution) were included between 2 successive targets. The time interval between successive tones, irrespective of whether they were targets or standard tones, ranged between 2 and 3 seconds, distributed uniformly. Consequently, the minimum inter-target interval was 8 s. The number of trials was adjusted so that the total duration of the task was 30 min. The patients were installed in front of a computer screen with a grey background and were instructed to fixate on a white cross in the center of the screen. Task performance was evaluated by measuring the time between each target onset and the following response (reaction time, RT). The data was separated in 10 equally-sized epochs, and the mean of the RT was computed within each epoch, as well as for the entire experiment (RT mean). We then evaluated the progression of the RT over time by computing the Spearman correlation between the epoch index and the corresponding average RT (RT change).

### Pupillometry and pupil data analysis

We recorded pupil size continuously during the oddball detection task. Given the assumed link between LC and pupil response^29^, this measure was acquired in order to obtain, in addition to the structural measure provided by the neuromelanin sequence, an estimate of the functional integrity of the noradrenergic system. We used the Eyelink 1000 eyetracker (SR Research, Ottawa, Canada), situated just below the screen and centered on the dominant eye of the participant. The 500 Hz pupil size signal was then normalized, downsampled to 10 Hz and analyzed by means of an autoregressive model with exogenous inputs (ARX^45^). This method allowed us to isolate the pupillary response to distractors, targets and blinks. Given the non-stationarity of the pupil signal during prolonged recordings, which violated the assumption of the ARX method, we sliced the 30-minute data into six 5-minute chunks. Because of this additional block variable, we analyzed pupil responses with linear mixed models (fitlme in Matlab; model: Pupil~SN + LC + Block + (Block|Subject)).

### MRI data acquisition

All subjects were scanned using 3 T scanner (Achieva, Philips Healthcare, Eindhoven, the Netherlands) with a SENSE 32-channel phased array head coil. Neuromelanin-sensitive images were obtained using T1 turbo spin-echo sequence: FOV 220 × 220 mm^2^, 16 slices aligned perpendicularly to the commissural-obex plane, 0.43 × 0.43 × 2.5 mm resolution, TR/TE/FA = 600 ms/14 ms/90°. We also acquired whole brain Turbo Field Echo anatomical images with the following parameters: FOV 220 × 197 mm^2^, 150 slices, 0.81 × 0.95 × 1 mm resolution (reconstruction 0.75 × 0.75 × 1), TR/TE/FA = 9.1 ms/4.6 ms/8°.

### LC mask alignment

The MRI data was analyzed using SPM8 (Wellcome Department of Imaging Neuroscience, London, UK; www.fil.ion.ucl.ac.uk/spm) and in-house developed MATLAB code. The normalization consisted first in aligning the individual neuromelanin-sensitive and whole-brain anatomical images and then normalizing the whole-brain anatomical image to the atlas template. Having established the mapping between neuromelanin-sensitive, whole-brain and atlas images, we used the corresponding transformation parameters to project the location of LC defined in atlas space^46^ to the individual neuromelanin-sensitive image. This approach resulted in only approximate positions of LC due to the idiosyncrasies of individual brain anatomy and inevitable imperfections of the normalization procedure. Therefore, we performed an intensity-driven alignment by shifting the atlas masks derived from the normalization step to the position with the highest average signal intensity. The space of possible shifts was defined in all three axes. Inward-outward shifts between left and right LCs was also permitted, maximal x – y – z – in/out: 4–4–2–2 voxels (Supplementary Fig. 1). The shifts were smaller in z axis because slice thickness was considerably bigger than in-plane voxel size (2.5 vs 0.4 mm). Following the alignment, we used K-means algorithm to split all voxels within the mask into K clusters, based on signal intensity. K-means iteratively assigns each data point to one of the K clusters, minimizing the sum of squared distances from the data point to cluster mean. The value of K has to be chosen *a priori*, and we performed the correlations with a range of K values between 2 and 4. Since results did not depend on K, we finally used the LC metrics based on 2 clusters for the sake of brevity. SN degeneration was quantified with an identical procedure, following initial isolation based on probabilistic SN atlas^47^ (Supplementary Fig. 2).

## Electronic supplementary material

Supplementary information

## References

[1] Friedman, J. H. *et al*. Fatigue in Parkinson’s disease: report from a mutidisciplinary symposium, 10.1038/npjparkd.2015.25 (2016). 10.1038/npjparkd.2015.25 PMC4883681 27239558

[2] Shulman, L. M., Taback, R. L., Rabinstein, A. A. & Weiner, W. J. Non-recognition of depression and other non-motor symptoms in Parkinson’ s disease. **8**, 193–197 (2002). 10.1016/s1353-8020(01)00015-3 12039431

[3] Sara SJ (2009). The locus coeruleus and noradrenergic modulation of cognition. Nat. Rev. Neurosci..

[4] Carter ME (2010). Tuning arousal with optogenetic modulation of locus coeruleus neurons. Nat. Neurosci..

[5] Berridge, C. W. & Waterhouse, B. D. The locus coeruleus – noradrenergic system: modulation of behavioral state and state-dependent cognitive processes. **42**, 33–84 (2003). 10.1016/s0165-0173(03)00143-7 12668290

[6] Chan-palay. Alterations in Catecholamine Neurons of the Locus Coeruleus in Senile Dementia of the Ahheher Type and in Parkinson’s Disease With and Without Dementia and Depression **392** (1989). 10.1002/cne.902870308 2570794

[7] Taquet (1982). Microtopography of Methionine-Enkephalin, Dopamine and Noradrenaline in The Ventral Mesencephalon of Humancontrol And Parkinsonian Brains. Tissue Prep..

[8] Del Tredici K, Rüb U, De Vos RAI, Bohl JRE, Braak H (2002). Where does Parkinson disease pathology begin in the brain?. J. Neuropathol. Exp. Neurol..

[9] García-lorenzo, D., Santos, C. L., Ewenczyk, C. & Leu-semenescu, S. The coeruleus/subcoeruleus complex in rapid eye movement sleep behaviour disorders in Parkinson’ s disease. **136**, 2120–2129 (2013). 10.1093/brain/awt152 PMC3692035 23801736

[10] Ehrminger, M. *et al*. The coeruleus/subcoeruleus complex in idiopathic rapid eye movement sleep behaviour disorder. 1180–1188, 10.1093/brain/aww006 (2016). 10.1093/brain/aww006 26920675

[11] Delaville, C., Deurwaerdère, P. D & Benazzouz, A. Noradrenaline and Parkinson’s Disease. *Front. Syst. Neurosci*. **5** (2011). 10.3389/fnsys.2011.00031 PMC3103977 21647359

[12] Shibata E (2008). Use of Neuromelanin-Sensitive MRI to Distinguish Schizophrenic and Depressive Patients and Healthy Individuals Based on Signal Alterations in the Substantia Nigra and Locus Ceruleus. Biol. Psychiatry.

[13] Remy P, Doder M, Lees A, Turjanski N, Brooks D (2005). Depression in Parkinson’s disease: loss of dopamine and noradrenaline innervation in the limbic system. Brain.

[14] McNamara P, Durso R (2006). Neuropharmacological treatment of mental dysfunction in Parkinson’s disease. Behavioural Neurology.

[15] Aston-Jones, G., Rajkowski, J., Kubiak, P. & Alexinsky, T. Locus Coeruleus Neurons in Monkey Are Selectively Activated by Attended Cues in a Vigilance Task. *J. Neurosci*., 10.1523/JNEUROSCI.14-07-04467.1994 (1994). 10.1523/JNEUROSCI.14-07-04467.1994 PMC6577022 8027789

[16] Schifitto G (2008). Fatigue in levodopa-naive subjects with Parkinson disease. Neurology.

[17] Chou KL, Kotagal V, Bohnen NI (2016). Neuroimaging and clinical predictors of fatigue in Parkinson disease. Park. Relat. Disord..

[18] Pavese, N., Metta, V., Bose, S. K., Chaudhuri, K. R. & Brooks, D. J. Fatigue in Parkinson’ s disease is linked to striatal and limbic serotonergic dysfunction. 3434–3443, 10.1093/brain/awq268 (2010). 10.1093/brain/awq268 20884645

[19] Loebel M (2016). Antibodies to β adrenergic and muscarinic cholinergic receptors in patients with Chronic Fatigue Syndrome. Brain. Behav. Immun..

[20] Barone, P. *et al*. The Priamo Study: A Multicenter Assessment of Nonmotor Symptoms and Their Impact on Quality of Life in Parkinson’ s Disease. **24**, 1641–1649 (2009). 10.1002/mds.22643 19514014

[21] Franssen M, Winward C, Collett J, Wade D, Dawes H (2014). Interventions for fatigue in Parkinson’s disease: A systematic review and meta-analysis. Mov. Disord..

[22] Papakostas GI (2006). Resolution of Sleepiness and Fatigue in Major Depressive Disorder: A Comparison of Bupropion and the Selective Serotonin Reuptake Inhibitors. Biol. Psychiatry.

[23] Kumar S, Bhatia M, Behari M (2002). Sleep disorders in Parkinson’s disease. Mov. Disord..

[24] van Hilten JJ (1993). Sleep, excessive daytime sleepiness and fatigue in Parkinson’s disease. J. Neural Transm. Park. Dis. Dement. Sect..

[25] Benoit, C.-E. *et al*. Cognitive task avoidance correlates with fatigue-induced performance decrement but not with subjective fatigue. *Neuropsychologia*, 10.1016/j.neuropsychologia.2018.06.017 (2018). 10.1016/j.neuropsychologia.2018.06.017 29936122

[26] Baker KG, Törk I, Hornung JP, Halasz P (1989). The human locus coeruleus complex: an immunohistochemical and three dimensional reconstruction study. Exp. brain Res..

[27] Manaye KF, McIntire DD, Mann DMA, German DC (1995). Locus coeruleus cell loss in the aging human brain: A non-random process. J. Comp. Neurol..

[28] Frenklach A (2016). Management of Depression in Parkinson’s Disease. Am. J. Psychiatry Resid. J..

[29] Aston-Jones G, Cohen JD (2005). An Integrative Theory of Locus Coeruleus-Norepinephrine Function: Adaptive Gain and Optimal Performance. Annu. Rev. Neurosci..

[30] Joshi S, Li Y, Kalwani RM, Gold JI (2016). Relationships between Pupil Diameter and Neuronal Activity in the Locus Coeruleus, Colliculi, and Cingulate Cortex. Neuron.

[31] Szabadi E (2013). Functional neuroanatomy of the central noradrenergic system. J. Psychopharmacol..

[32] Varazzani C, San-Galli A, Gilardeau S, Bouret S (2015). Noradrenaline and Dopamine Neurons in the Reward/Effort Trade-Off: A Direct Electrophysiological Comparison in Behaving Monkeys. J. Neurosci..

[33] Briand LA, Gritton H, Howe WM, Young DA, Sarter M (2007). Modulators in concert for cognition: modulator interactions in the prefrontal cortex. Prog. Neurobiol..

[34] Sulzer D (2018). Neuromelanin detection by magnetic resonance imaging (MRI) and its promise as a biomarker for Parkinson’s disease. npj Park. Dis..

[35] Castellanos G (2015). Automated Neuromelanin Imaging as a Diagnostic Biomarker for Parkinson’s Disease. Mov. Disord..

[36] Ohtsuka C (2014). Differentiation of early-stage parkinsonisms using neuromelanin-sensitive magnetic resonance imaging. Park. Relat. Disord..

[37] Schwarz, S. T. *et al*. Parkinson’s disease related signal change in the nigrosomes 1–5 and the substantia nigra using T2* weighted 7T MRI. *NeuroImage Clin*., 10.1016/j.nicl.2018.05.027 (2018). 10.1016/j.nicl.2018.05.027 PMC5986169 29872633

[38] Isaias, I. U. *et al*. Neuromelanin imaging and dopaminergic loss in parkinson’s disease. *Front. Aging Neurosci*. **8** (2016). 10.3389/fnagi.2016.00196 PMC4992725 27597825

[39] Diedrichsen J (2006). A spatially unbiased atlas template of the human cerebellum. Neuroimage.

[40] Goetz CG (2008). Movement Disorder Society-sponsored revision of the Unified Parkinson’s Disease Rating Scale (MDS-UPDRS): scale presentation and clinimetric testing results. Mov. Disord..

[41] Brown RG, Dittner a, Findley L, Wessely SC (2005). The Parkinson fatigue scale. Park. Relat. Disord..

[42] Beck, A. T., Steer, R. A. & Brown, G. K. Manual for the Beck depression inventory-II. *San Antonio, TX Psychol. Corp*. 1–82 (1996).

[43] Trenkwalder C (2011). Parkinson’s disease sleep scale–validation of the revised version PDSS-2. Mov. Disord..

[44] Tomlinson CL (2010). Systematic Review of Levodopa Dose Equivalency Reporting in Parkinson’ s Disease..

[45] Zénon A (2017). Time-domain analysis for extracting fast-paced pupil responses. Sci. Rep..

[46] Keren NI, Lozar CT, Harris KC, Morgan PS, Eckert MA (2009). *In vivo* mapping of the human locus coeruleus. Neuroimage.

[47] Keuken MC (2014). Quantifying inter-individual anatomical variability in the subcortex using 7T structural MRI. Neuroimage.

