# Supplementary material for: Locus Coeruleus atrophy doesn’t relate to fatigue in Parkinson’s disease
Source: Sci Rep. 2018 Aug 17;8:12381. doi: 10.1038/s41598-018-30128-y (PMC6098016; doi:10.1038/s41598-018-30128-y)
Supplement: Supplementary file 1 — Supplementary information [file 41598_2018_30128_MOESM1_ESM.docx]

# Locus Coeruleus atrophy doesn’t relate to fatigue in Parkinson’s disease

Oleg Solopchuk^1,3,*^, Moustapha Sebti^1^, Céline Bouvy^2^, Charles-Etienne Benoit^1^, Thibault Warlop^2^, Anne Jeanjean^2^ and Alexandre Zénon^1,3^

^1^ Institute of Neuroscience, Université catholique de Louvain, Brussels, Belgium

^2^ Cliniques Universitaires Saint Luc, Université catholique de Louvain, Brussels, Belgium

^3^ INCIA, 33076 Bordeaux, France

** Corresponding autho*r:

*Institute of Neuroscience,*

*Université catholique de Louvain,*

*53, Avenue Mounier*

*COSY- B1.53.04S*

*1200 Brussels, Belgium.*

E-mail: [oleg.solopchuk@uclouvain.be](mailto:oleg.solopchuk@uclouvain.be)

| **Sex** | 21 M, 17 F | **UPDRS** **Ia** (/24) | 3.469 ± 3.37 |
| --- | --- | --- | --- |
| **Age** (years) | 61.45 ± 10.14 | **UPDRS Ib** (/32) | 8.774 ± 4.46 |
| **Duration** (years) | 6.52 ± 5.99 | **UPDRS II** (/52) | 12.29 ± 6.19 |
| **LED** (mg) | 796.96 ± 470.04 | **UPDRS III** (/132) | 25.40 ± 14.85 |
| **PFS** (/80) | 46.87 ± 14.61 | **UPDRS IV** (/24) | 3.84 ± 3.77 |
| **BDI** (/39) | 12.46 ± 7.56 | **UPDRS** total (/260) | 53.09 ± 23.48 |
| **PDSS** (/60) | 16.86 ± 9.65 | **H & Y** (/5) | 1.93 ± 0.69 |

Supplementary Table 1. *Clinical characteristics of the subjects. MDS-UPDRS consists of several parts: I - non- motor experiences of daily living, II - motor experiences of daily living, III - motor examination, IV - motor complications. Only the total score was used in the analyses.*


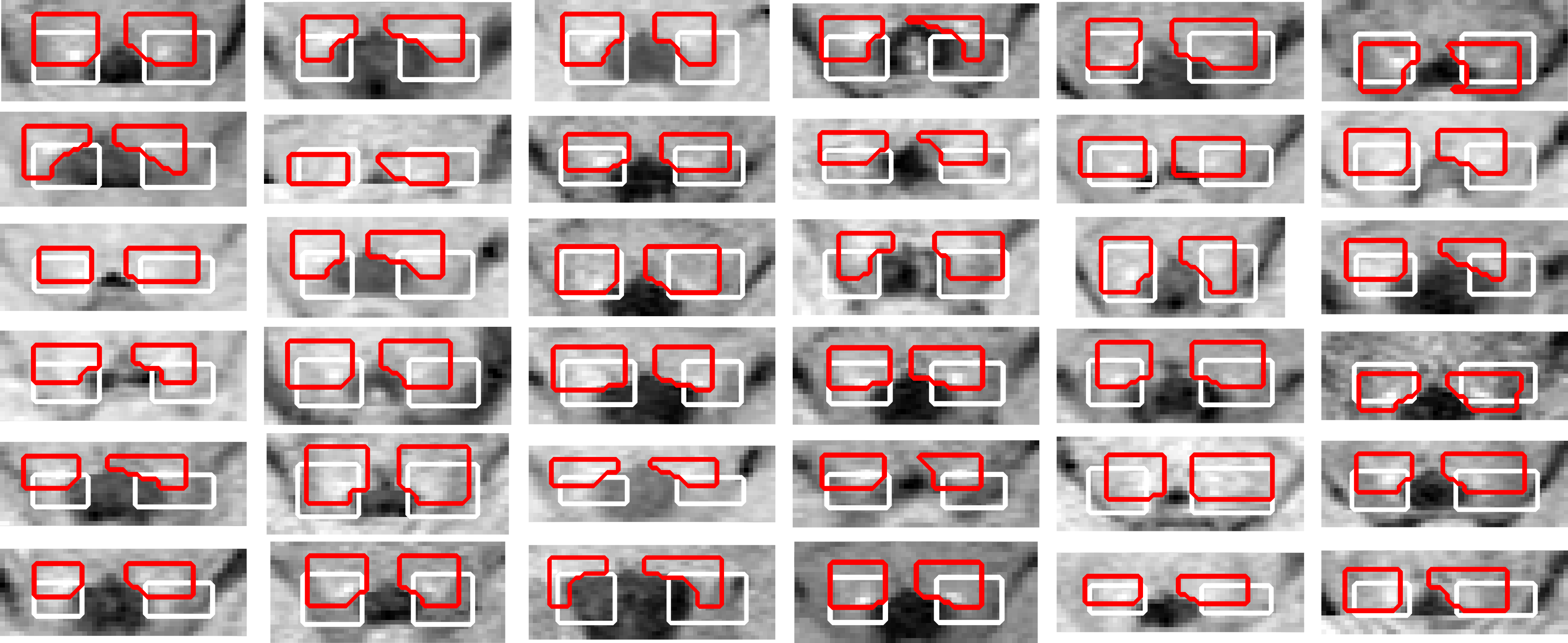


Supplementary Figure 1. *LC masks for all 36 subjects (only middlemost slice shown; white mask – atlas aligned on the basis of stereotactic coordinates, red – following fine tuning with in-house algorithm, see Methods).*


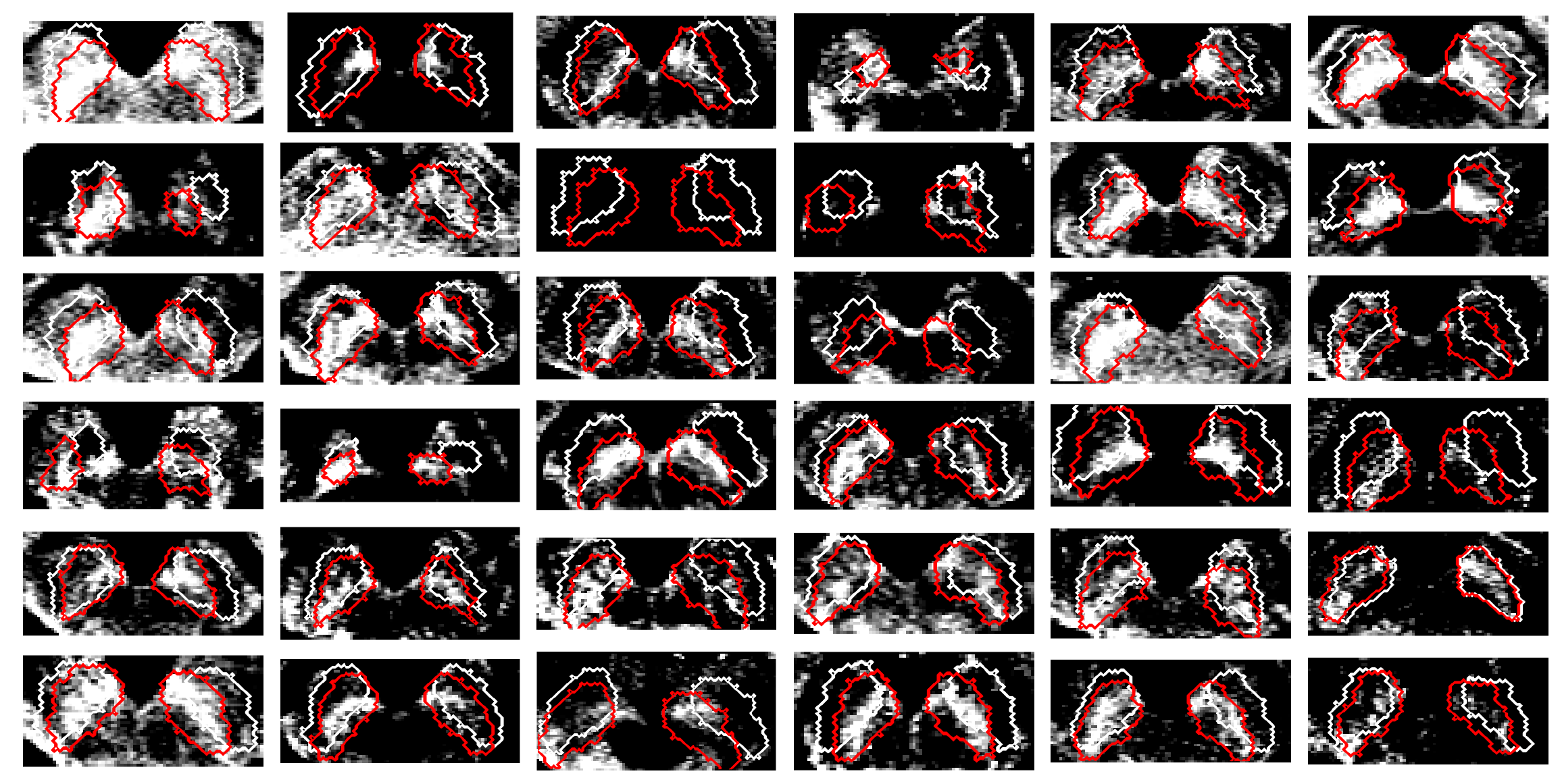


Supplementary Figure 2. *SN masks for all 36 subjects (only middlemost slice shown; white mask – atlas aligned on the basis of stereotactic coordinates, red – following fine tuning with in-house algorithm, see Methods).*


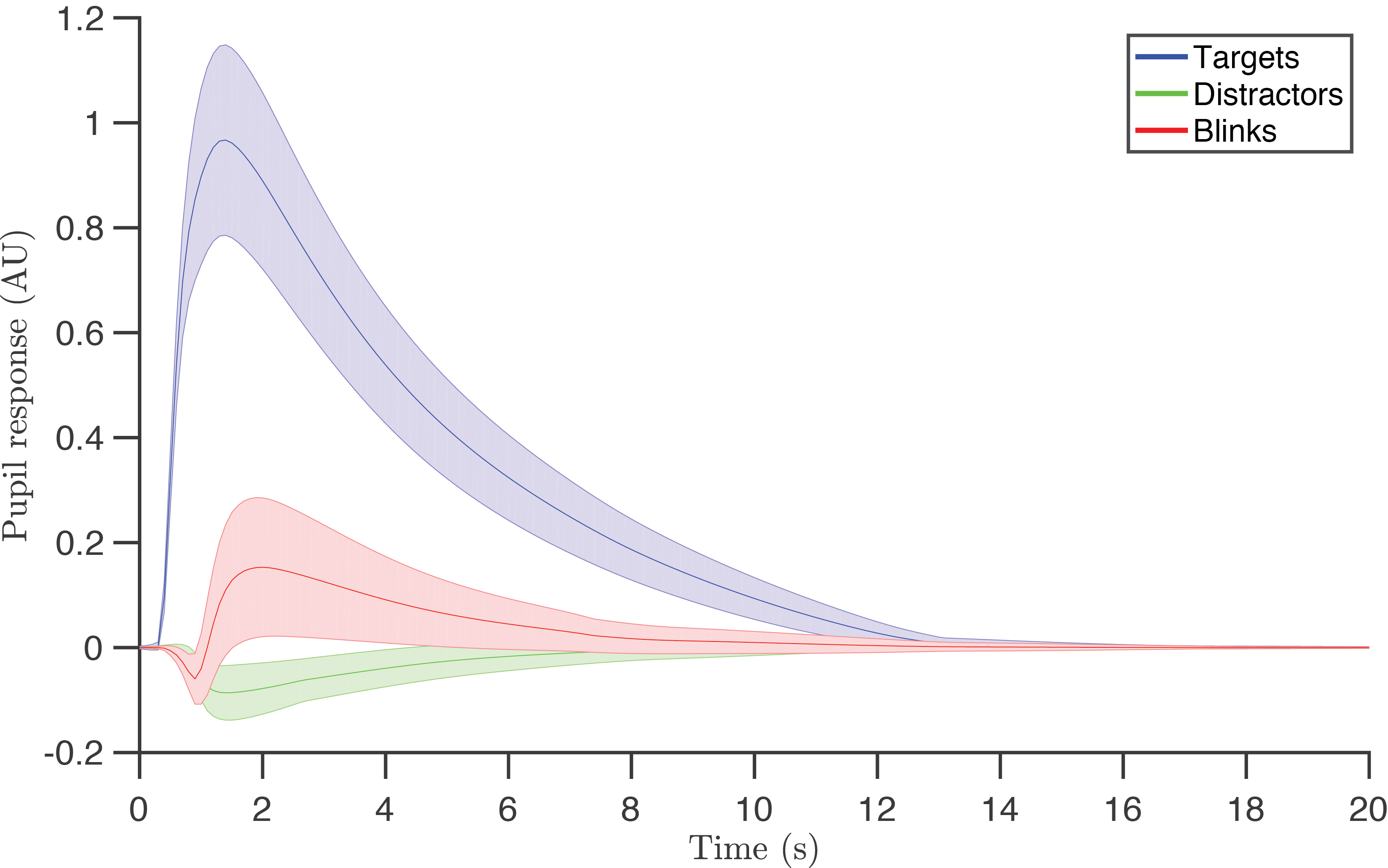


Supplementary Figure 3. *Average across subjects of the isolated pupillary impulse responses to targets, distractors and blinks, obtained by means of the ARX method. The data shown on the figure included the whole 30-minute session, with the shaded area illustrating standard error of the mean. Statistical analyses were performed on 5-minute blocks to avoid non-stationarity issues.*
